# Role of uL3 in the Crosstalk between Nucleolar Stress and Autophagy in Colon Cancer Cells

**DOI:** 10.3390/ijms21062143

**Published:** 2020-03-20

**Authors:** Annalisa Pecoraro, Pietro Carotenuto, Brunella Franco, Rossella De Cegli, Giulia Russo, Annapina Russo

**Affiliations:** 1 Department of Pharmacy, University of Naples “Federico II”, Via Domenico Montesano 49, 80131 Naples, Italy; annalisa.pecoraro@unina.it; 2 Telethon Institute of Genetics and Medicine (TIGEM), Via Campi Flegrei, 34, 80078 Pozzuoli, Naples, Italy; p.carotenuto@tigem.it (P.C.); franco@tigem.it (B.F.); decegli@tigem.it (R.D.C.); 3 The Institute of Cancer Research, Cancer Therapeutics Unit 15 Cotswold Road, Sutton, London SM2 5NG, UK; 4 Medical Genetics, Department of Translational Medical Sciences, University of Naples “Federico II”, Via Sergio Pansini 5, 80131 Naples, Italy

**Keywords:** cancer, chemotherapy, nucleolar stress, nucleolus, p53, ribosomal proteins, uL3, autophagy

## Abstract

The nucleolus is the site of ribosome biogenesis and has been recently described as important sensor for a variety of cellular stressors. In the last two decades, it has been largely demonstrated that many chemotherapeutics act by inhibiting early or late rRNA processing steps with consequent alteration of ribosome biogenesis and activation of nucleolar stress response. The overall result is cell cycle arrest and/or apoptotic cell death of cancer cells. Our previously data demonstrated that ribosomal protein uL3 is a key sensor of nucleolar stress activated by common chemotherapeutic agents in cancer cells lacking p53. We have also demonstrated that uL3 status is associated to chemoresistance; down-regulation of uL3 makes some chemotherapeutic drugs ineffective. Here, we demonstrate that in colon cancer cells, the uL3 status affects rRNA synthesis and processing with consequent activation of uL3-mediated nucleolar stress pathway. Transcriptome analysis of HCT 116^p53−/−^ cells expressing uL3 and of a cell sub line stably depleted of uL3 treated with Actinomycin D suggests a new extra-ribosomal role of uL3 in the regulation of autophagic process. By using confocal microscopy and Western blotting experiments, we demonstrated that uL3 acts as inhibitory factor of autophagic process; the absence of uL3 is associated to increase of autophagic flux and to chemoresistance. Furthermore, experiments conducted in presence of chloroquine, a known inhibitor of autophagy, indicate a role of uL3 in chloroquine-mediated inhibition of autophagy. On the basis of these results and our previous findings, we hypothesize that the absence of uL3 in cancer cells might inhibit cancer cell response to drug treatment through the activation of cytoprotective autophagy. The restoration of uL3 could enhance the activity of many drugs thanks to its pro-apoptotic and anti-autophagic activity.

## 1. Introduction

Colon cancer is the most common cause of cancer-related death in the development countries. Treatment involves therapeutic application of chemotherapeutic agents associated to surgical therapy [1]. However, the clinical outcome of this therapeutic approach can be unsatisfactory especially in patients with advanced disease or in presence of metastasis. Furthermore, common chemotherapeutic drugs show high levels of toxicity for normal cells and development of chemoresistence [2]. Therefore, many attempts have been made to find a best combination of chemotherapeutic agents with little toxicity on normal cells and to better understand the molecular basis underlying the mechanism of chemoresistance [3]. 

It has been demonstrated that most chemotherapeutic drugs used in colon cancer are able to activate the so-called nucleolar stress response which in turn causes translocation of ribosomal proteins (r-proteins) as ribosome free proteins from the nucleolus to the nucleoplasm [4,5]. The nucleoplasmatic accumulation of these proteins is associated, through mechanisms dependent or independent of p53, to cell cycle arrest and/or apoptosis [4]. 

Recently, a large number of studies conducted by our group focused on post-transcriptional regulation of r-protein synthesis [6,7,8] and on extra-ribosomal functions of some r-proteins [4]. In particular, we demonstrated that r-protein uL3 is a key mediator of nucleolar stress induced by several chemotherapeutic drugs as 5-fluorouracil (5-FU), Oxaliplatinum (OHP), Actinomycin D (Act D) and Niclosamide in p53-mutated lung and p53-deleted colon cancer cells [9,10,11,12]. Specifically, we identified a new nucleolar stress pathway activated upon cell treatment with chemotherapeutic drugs that is p53-independent and uL3-dependent [4]. More important, we demonstrated that the expression of uL3 is downregulated in colon tumor tissues [13] and that uL3 overexpression stimulated apoptotic cell death by inducing late apoptosis [14]. Combined treatment with 5-FU plus uL3 resulted more effective in inducing apoptosis in cancer cells than 5-FU or uL3 alone [13,15].

Data from the literature largely demonstrate that some r-proteins have a central role in the development of chemoresistance [16]. Recently, we have demonstrated that uL3 is involved in acquired resistance of cancer cells to common chemotherapeutic drugs [17].

Autophagy has been linked to both tumorigenesis and chemotherapeutic resistance [18]. In physiological condition, cells utilize autophagy to control cell contents, to eliminate old proteins and damage organelles for the maintenance of homeostasis [19]. In tumors, the autophagic process can either help to enhance or to resist apoptosis, depending on the cell context [20]. Apoptosis and autophagy are both activated by similar stressors including chemotherapeutics. Although some anticancer treatments can induce autophagic cell death, many recent studies have concluded that in tumors, autophagy works as a prosurvival process that eliminates damaged organelles and recycles macromolecules in response to metabolic stresses induced by anticancer drugs [21]. In this way, autophagy has emerged as mechanism of chemoresistance. In this context, the inhibition of autophagy can re-sensitize resistant cancer cells, enhancing the effectiveness of anticancer agents [22]. 

Here, we demonstrate that silencing of uL3 in colon cancer cells causes the induction of autophagy associated to Act D resistance, while the rescue of uL3 associated to the inhibition of autophagy with consequent re-sensitization of cancer cells to drug treatment. In the light of these data, we conclude that uL3 can enhance the pro-apoptotic activity of many drugs thanks to its pro-apoptotic and anti-autophagic activity. 

This study presents new insights into our understanding of the link between drug inducing nucleolar stress and autophagy, and provides a potential therapeutic strategy for the management of tumors lacking functional p53 and having decreased levels of uL3.

## 2. Results

### 2.1. Alteration of uL3 Intracellular Levels Causes Defects in rRNA Processing

Starting from the notion that uL3 plays a key role in cell response to nucleolar stress pathways activated by chemotherapeutic drugs we wondered whether uL3 might *per se* elicit nucleolar stress pathway through impairing of ribosomal gene processing as already demonstrated for other r-proteins [4]. 

**Figure 1:**
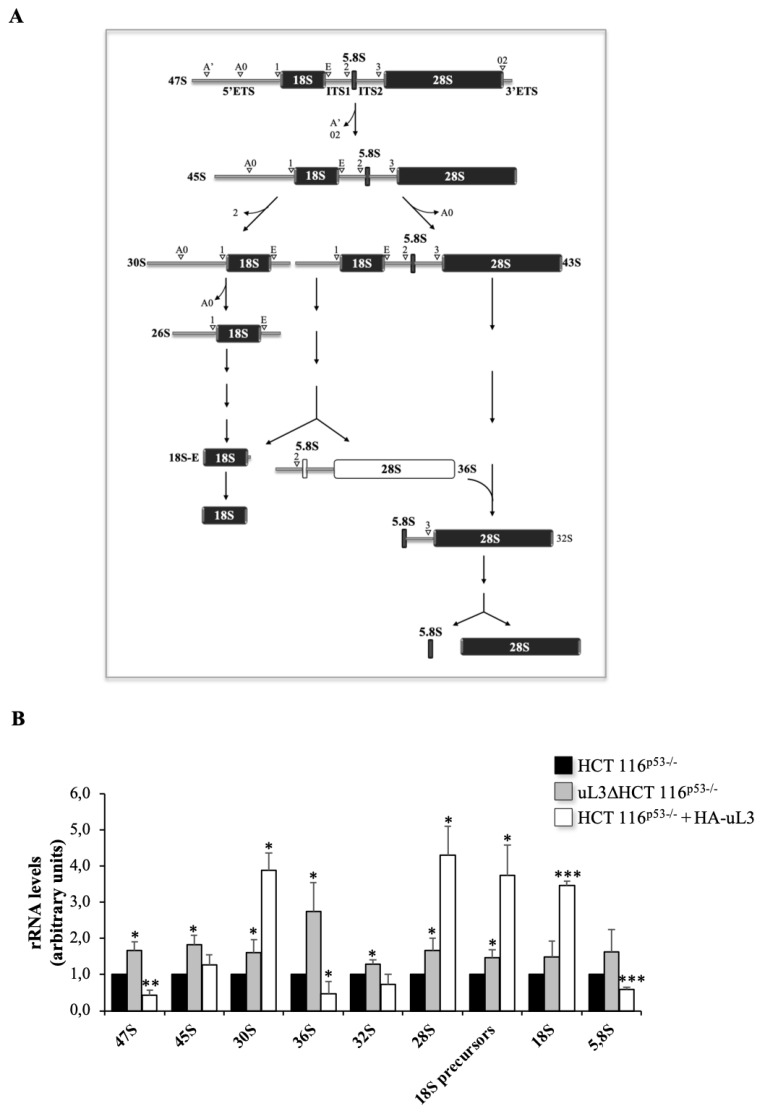
Enforced expression of uL3 affects rRNA processing. (**A**) Schematic representation of rRNA maturation process. Cleavage sites are indicated with white arrows. (**B**) Total RNA from HCT 116^p53−/−^ cells transfected or not with 1 µg of pHA-uL3 and uL3ΔHCT 116^p53−/−^ cells was subjected to RT-qPCR with primers specific for intermediates and mature rRNAs (Table 1). Quantification of signals is shown. Bars represent the mean of triplicate experiments; error bars represent the standard deviation. Untreated cells was set at 1. * *p* < 0.05, ** *p* < 0.01, *** *p* < 0.001 vs. HCT 116^p53−/−^ cells set at 1.

Within the nucleolus, ribosomal genes are transcribed by RNA polymerase I (Pol I) to produce the 47S rRNA precursor, a single transcript that is then cleaved and processed to generate the mature 28S, 18S and 5.8S rRNAs. In 47S rRNA precursor, the mature rRNAs are flanked by non-coding spacer sequences, which include the 5′ and 3′ external transcribed spacers (ETS) and internal transcribed spacers (ITS) 1 and 2. These transcribed spacers contain several cleavage sites and are gradually eliminated by the sequential action of endo- and exo-ribonucleases schematically reported in Figure 1A [23]. 

To understand the role of uL3 on rRNA processing, we analyzed the production of rRNA precursors and rRNA mature transcripts upon alteration of uL3 expression. 

To this aim, total RNA was extracted from HCT 116^p53−/−^ cells, HCT 116^p53−/−^ transiently transfected with pHA-uL3 and uL3ΔHCT 116^p53−/−^, a cell subline stably silenced of uL3 [10], and the relative aboundance of intermediates and mature rRNA transcripts was determined by RT-qPCR with specific primers (Table 1). Transfection efficiency of HA-uL3 was analyzed by Western blotting (Figure S1).

Enforced expression of uL3 caused a reduction of about 60% of the 47S primary transcript while in absence of uL3 the production of this transcript increased (Figure 1B).

In order to better characterize the role of uL3 in pre-rRNA processing we analyzed the level of other rRNA products. The initial cleavage of the 47S pre-rRNA at sites A’ in the 5′-ETS and 02 in the 3′-ETS led to the production of 45S pre-rRNA (Figure 1A). As shown in Figure 1B, HCT 116^p53−/−^ cells overexpressing uL3 accumulated 45S transcript. This pre-rRNA is further processed either by cleavage of the 5′ETS or by elimination of the ITS1 at site 2 to produce mature rRNAs (Figure 1A). Of interest, higher levels of uL3 resulted in a strong accumulation of 30S pre-rRNA which was associated to increased production of its corresponding mature transcript, 18S rRNA. In this condition, the other two mature rRNAs were also affected by higher intracellular amount of uL3. In fact, 28S rRNA accumulated while 5,8S rRNA reduced. Alteration in rRNA processing was also observed in cells stably deleted of uL3. In particular, deletion of uL3 caused a significant enrichment of 36S pre-rRNA (Figure 1B). 

The presence of 36S pre-rRNA, that is normally expressed at low levels in normal cells, is due to the inhibition of processing at site 2 within ITS1 and could be responsible for the activation of nucleolar stress response in treated cells. The accumulation of this characteristic transcript might not affect the subsequent maturation of the 5,8S and 28S rRNA [24]. In fact, levels of 28S, 18S and 5,8S rRNAs resulted only slightly affected compared with control, HCT 116^p53−/−^ cells (Figure 1B).

These results indicate that uL3 status is crucial for correct rRNA processing.

### 2.2. Effect of uL3 Status on 47S pre-rRNA Synthesis and Stability

**Figure 2:**
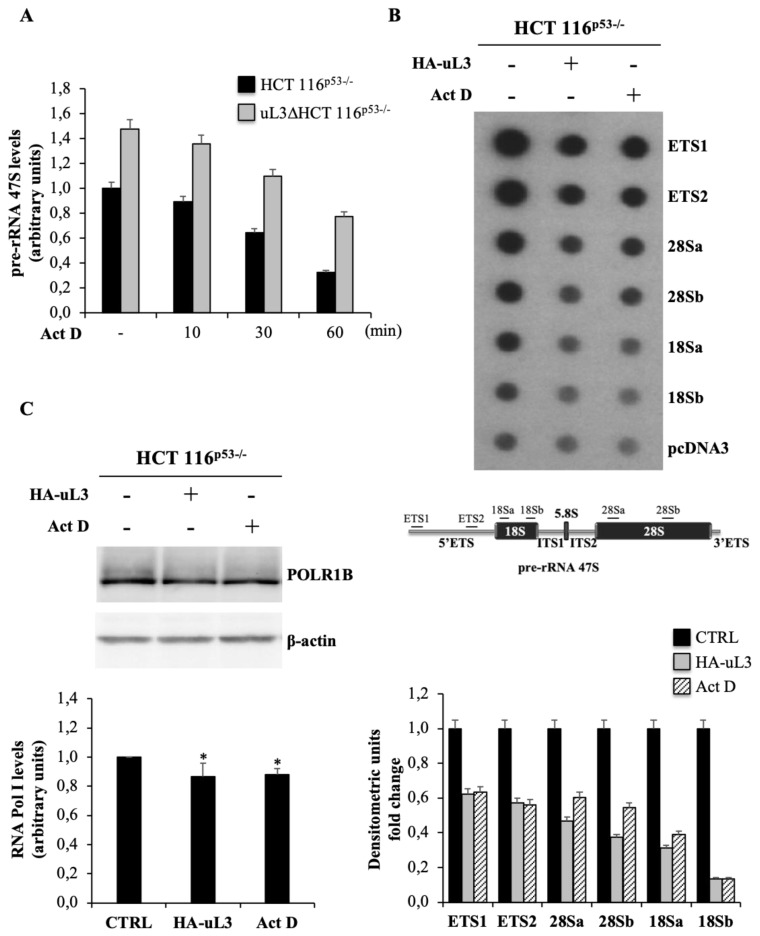
uL3 status affects 47S pre-rRNA synthesis and stability. (**A**) Total RNA from HCT 116^p53−/−^ and uL3ΔHCT 116^p53−/−^ cells, treated with Act D 5 nM for 0, 10, 30 and 60 min was subjected to RT-qPCR with primers specific for 5′ETS region of 47S pre-rRNA and β-actin mRNA (Table 1). Quantification of signals is shown. Bars represent the mean of triplicate experiments; error bars represent the standard deviation. (**B**) Nuclear run-on assay. 20 μg of plasmid DNA including sequences of two regions of 5′ETS, two regions of 28S, two regions of 18S and plasmid pcDNA3 were spotted on membrane and incubated with ^32^P-labeled RNA from untreated HCT 116^p53−/−^, HCT 116^p53−/−^ cells transfected with 1 µg pHA-uL3 and HCT 116^p53−/−^ cells treated with Act D. The average of two signals normalized for pHA-uL3 is reported in the bar graph in the lower panel. A schematic diagram of 47S pre-rRNA indicating the regions used in nuclear run-on assay is shown in the lower panel. Full-length blot is shown in Figure S5. (**C**) Total protein extracts from HCT 116^p53−/−^ cells untransfected or transfected with 1µg pHA-uL3 and HCT 116^p53−/−^ cells treated with Act D were analyzed by Western blot with the indicated antibodies. Full-length blots are shown in Figure S6. Quantification of signals is shown. Bars represent the mean of triplicate experiments; error bars represent the standard deviation. * *p* < 0.05 vs. HCT 116^p53−/−^ cells set at 1.

To investigate whether the observed reduction of 47S pre-rRNA in HCT 116^p53−/−^ cells overexpressing uL3 was associated to the alteration in rRNA synthesis or to the increase of 47S pre-rRNA turnover, we firstly monitored the rate of 47S pre-rRNA degradation in presence or in absence of uL3. To this aim, HCT 116^p53−/−^ and uL3ΔHCT 116^p53−/−^ cells were treated with low dose of Act D (5 nM), that acts at low concentrations as a specific inhibitor Pol I transcription, for 10, 30 and 60 min. Then, total RNA extracted from cell lysates was analyzed by RT-qPCR with specific primers for 47S pre-rRNA (Table 1). As shown in Figure 2A, analysis of rRNA amounts demonstrated that the status of uL3 did not affect the rate of rRNA degradation. In fact, we observed a reduction of rRNA levels of almost 10, 40 and 70% in both cell lines after 10, 30 and 60 min of treatment with Act D, respectively. This result suggests that the observed reduction of 47S pre-rRNA levels in HCT 116^p53−/−^ cells transfected with pHA-uL3 plasmid could reflect the inhibition of Pol I mediated transcription. To address this issue, we evaluated the rate of Pol I mediated transcription initiation by performing a nuclear run-on assay in cells upon overexpressing or deletion of uL3. The results of these experiments showed in Figure 2B indicate that in condition of uL3 overexpression Pol I mediated transcription activity was strictly reduced as with Act D treatment. As control, we measured the level of Pol I by Western blotting with specific antibodies in HCT 116^p53−/−^, HCT 116^p53−/−^ overexpressing uL3 and HCT 116^p53−/−^ treated with Act D. As shown in Figure 2C, Pol I level was not altered either in condition of uL3 over-expression then in condition of Act D treatment.

All together these data indicate that the over-expression of uL3 is associated to the inhibition of Pol I transcription with consequent alteration in rRNA processing and activation of uL3 mediated nucleolar stress response.

### 2.3. Identification of Genes and Pathways Differentially Expressed in HCT 116^p53−/−^ Cells in Presence or Absence of uL3

Using a transcriptomic RNA-seq analysis, we were thereafter interested to identify the transcripts showing differential expression levels between HCT 116^p53−/−^ and uL3ΔHCT 116^p53−/−^ cells, treated or not with Act D in order to better understand the role of uL3 in the activation of nucleolar stress pathway and in chemoresistance. We have previously demonstrated that the treatment of HCT 116^p53−/−^ cells with Act D induced a nucleolar stress pathway p53-independent but uL3-dependent. In fact, in condition of Act D treatment uL3 protein was up-regulated and as ribosome free form translocated to the nucleoplasm where it regulated key cellular processes such as cell cycle progression and apoptosis [11,12].

We collected RNA samples from HCT 116^p53−/−^ and uL3ΔHCT 116^p53−/−^ cells cultured upon Act D treatment and analyzed the data in order to reveal changes in gene expression between different replicates in specific experimental conditions.

**Figure 3:**
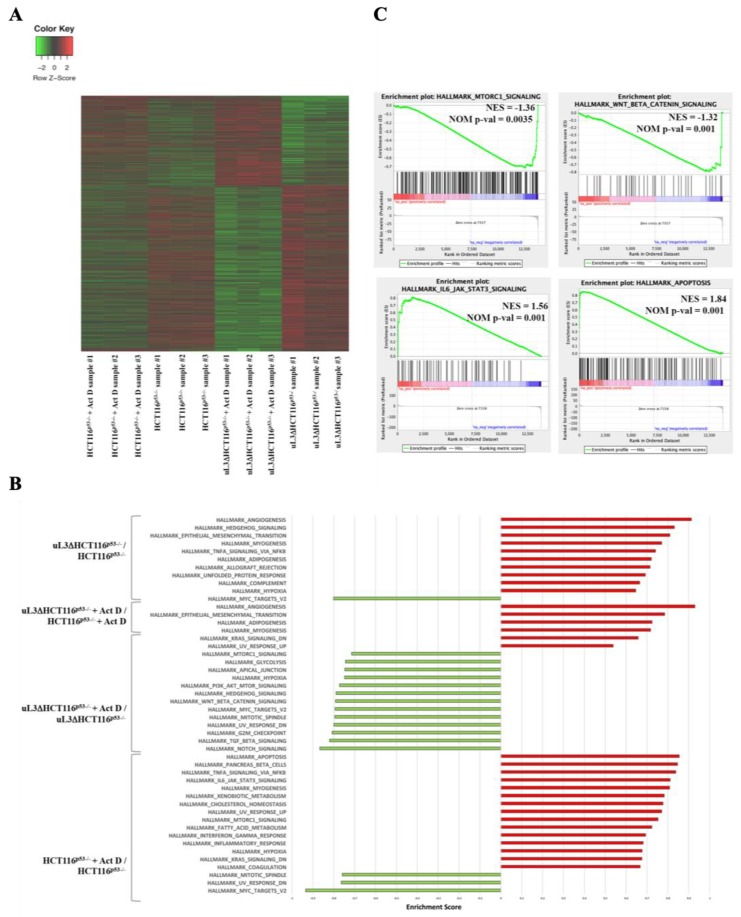
Key genes and pathways regulated by Act D treatment in HCT 116^p53−/−^ cells and uL3ΔHCT 116^p53−/−^ cells. (**A**) Unsupervised hierarchical Z-Score clustering. Clustering analyses revealed differentially expressed genes (DEGs) between the experimental groups. The color scale means the gene expression standard deviations from the mean, with green for low expression and red for the high expression levels. (**B**) GSEA querying hallmark genes depicting significant enrichment of signaling pathway genes as cell metabolism, apoptosis, autophagy, cell growth, as well as cancer-related pathways. (**C**) Enrichment plots for gene sets related to IL6/JAK/STAT3 and apoptosis pathways significantly enriched in Act D-treated HCT116^p53−/−^ cells versus untreated HCT116^p53−/−^ cells and for the most negatively enriched pathways mTORC 1 and WNT_Beta_Catenin in uL3ΔHCT 116^p53−/−^ cells after Act D treatment compared to deleted untreated cells. The top portion of each panel shows the normalized enrichment scores (NES) for each gene; the bottom portion of the plot indicates the value of the ranking metric moving down the list of ranked genes. Location of the gene set members are indicated by black lines in the center of the plot. The gray plot at the bottom represents the ranked list of all differentially expressed genes. Genes which are more highly expressed are in red whereas genes more highly expressed in blue. GSEA, Gene set enrichment analysis; NES, normalized enrichment score; FDR, false discovery rate.

The differentially expressed genes (DEGs) between HCT 116^p53−/−^ and uL3ΔHCT 116^p53−/−^ cells were 11268 (5735 down-regulated and 5533 up-regulated) as shown in Figure 3A. More specifically, a total of 1724 genes was found up-regulated and 2204 down-regulated in HCT 116^p53−/−^ cells, while 950 genes were significantly up-regulated and 1670 down-regulated by Act D treatment in uL3ΔHCT 116^p53−/−^ cells compared to vehicle (Figure 3A and Tables S1–S4).

To understand the crucial role played by uL3 in drug resistance, an unbiased pathway analysis was carried out by using gene sets that correspond to various hallmarks of defined biological states in the uL3-regulated transcriptome with or without Act D treatment. GSEA revealed a significant up-regulation of 10 gene set in uL3ΔHCT 116^p53−/−^ cells compared with parental cells (Figure 3B). Interestingly the GSEA analyses revealed the depletion of 1 gene set corresponding to MYC–target genes (Hallmark_Myc_Targets_V2, shown in Figure 3B). Four gene sets were found to be significatively upregulated in Act D-treated uL3ΔHCT 116^p53−/−^ cells versus the treated parental cells. Among them it is interesting to note the upregulation of 2 gene sets associated with tumour aggressiveness: Hallmark_Epithelial_Mesenkymal_Transition, Hallmark _Angiogenesis (Figure 3B and Figure S3 and Tables S5–S8).

The up-regulation of epithelial–mesenchymal transition (EMT) pathway, a migratory cellular program associated with tumor development and metastasis is in line with our previous data demonstrating that this pathway is activated in colon cancer cells in absence of uL3 [12]. A large number of gene sets were found significatively depleted (n = 13) in uL3ΔHCT 116^p53−/−^ after Act D treatment compared to untreated cells, while there were only two positively enriched (Figure 3B). It is particularly noteworthy that most negatively enriched pathways were related to autophagy activation: Hallmark_P13K_AKT_mTOR_Signaling, Hallmark_mTORC1_Signaling, Hallmark_Wnt_Beta_Catenin_Signaling (Figure 3C). The most up-regulated modules in HCT 116^p53−/−^ cells treated with Act D compared to untreated HCT 116^p53−/−^ cells consisted of positive regulator of apoptosis pathway, mTORC1 and IL6_JAK_STAT3 pathways (Figure 3C). The up-regulation of apoptosis pathway is in line with our previously results indicating a pro-apoptotic role for uL3, while the up-regulation of mTORC1 and IL6_JAK_STAT3 could be correlated with inhibition of autophagy uL3-mediated. The DEGs between of uL3ΔHCT 116^p53−/−^ and HCT 116^p53−/−^ cells treated with Act D, were reported in Tables S9 and S10.

Taken together these results suggest that the different behavior of HCT 116^p53−/−^ cells in presence or absence of uL3 should reflect at least in part a differential expression of genes related to the activation of autophagic pathway.

### 2.4. uL3 Status Affects the Autophagic Flux in Colon Cancer Cells

It has been largely accepted that autophagy is a mechanism for treatment failure in cancer [22].

On the basis of our previous findings [4,12,17], we hypothesized that uL3 might attenuate cancer cell response to drug treatment modulating autophagy.

To explore this hypothesis, we assessed the role of uL3 in autophagosome formation by two different assays.

Firstly, we examined the effect of uL3 on the distribution of LC3B and LAMP1, autophagic and lysosomal markers, respectively. To this aim, HCT 116^p53−/−^ and uL3∆HCT 116^p53−/−^ cells were subjected to confocal microscopy after immunofluorescence staining. Specifically, green fluorescence (LC3B) is associated with autophagic vesicles while red fluorescence (LAMP1) decorates lower-pH organelles such as autolysosomes. Autophagic flux increased when both green and red puncta were increased in cells, whereas autophagic flux was blocked when yellow puncta were increased and localised in perinuclear regions. HCT 116^p53−/−^ cells were transiently transfected with pHA-uL3 plasmid and 24 h later the levels of endogenous LC3B and LAMP1 were analyzed by confocal microscopy.

**Figure 4:**
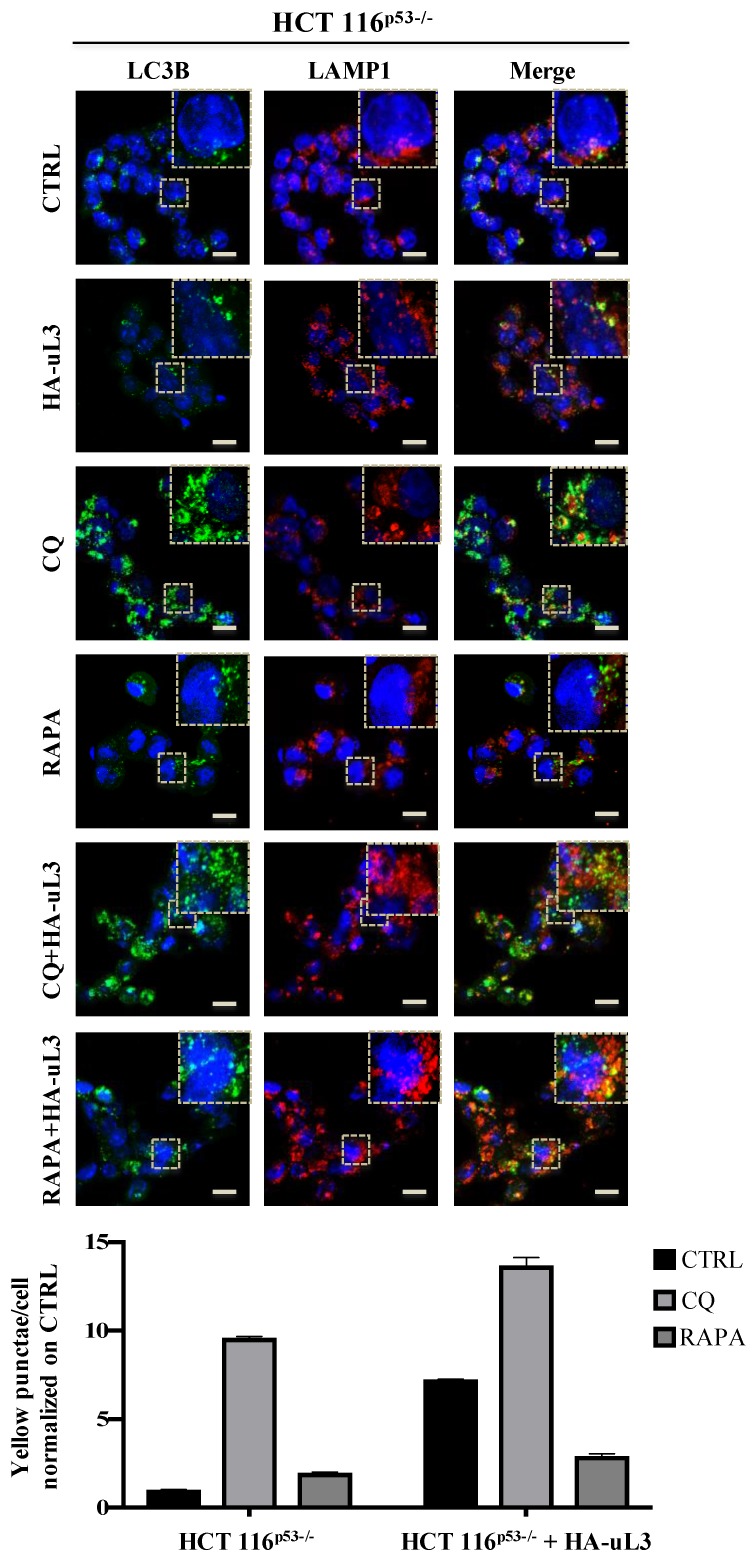
Enforced expression of uL3 inhibits autophagic flux in HCT 116^p53−/−^ cells. Representative z-stack images of HCT 116^p53−/−^ cells treated with 25 µM CQ or 1 µM RAPA or transiently transfected with 1 µg of pHA-uL3 for 24 h, alone or in combination. Cells were fixed and double stained with LC3B and LAMP1 antibodies. Nuclei were counterstained with Hoechst. Single-color fluorescence images of LC3B positive autophagosomes (green) and LAMP1 positive endosomes and/or lysosomes (red) are presented in the 1st and 2nd columns, respectively, and a merged view of these 2 proteins is shown in the 3rd column. Higher magnification views of the boxed area from the merged images are shown. Scal bar, 10 μm.

Figure 4 shows that upon the enforced expression of uL3 in the overlapping of images (LC3B-LAMP1, yellow) an increase of yellow punctate structures around the nucleus compared to control, untreated cells, was observed indicating a role of uL3 in inhibiting autophagy. In particular, the overexpression of uL3 was associated to increased autophagic vacuoles content (red).

These results are in line with the above reported bioinformatic analysis and suggest uL3 as inhibitory factor of autophagic flux.

The effect caused by uL3 on autophagy was further investigated in the presence of Rapamycin (RAPA), a well known inhibitor of oncogene mTOR [25] and activator of autophagy, and Chloroquine (CQ), which preventing the fusion of autophagosomes and lysosomes inhibits autophagy [26].

In HCT 116^p53−/−^ cells overexpressing uL3, RAPA failed to exert its effect. In fact, in this condition the number of yellow puncta increased compared to control, untreated cells, and to cells treated with RAPA alone (Figure 4).

In HCT 116^p53−/−^ cells transfected with pHA-uL3 and treated with CQ an increase of autophagy inhibition was observed (Figure 4).

**Figure 5:**
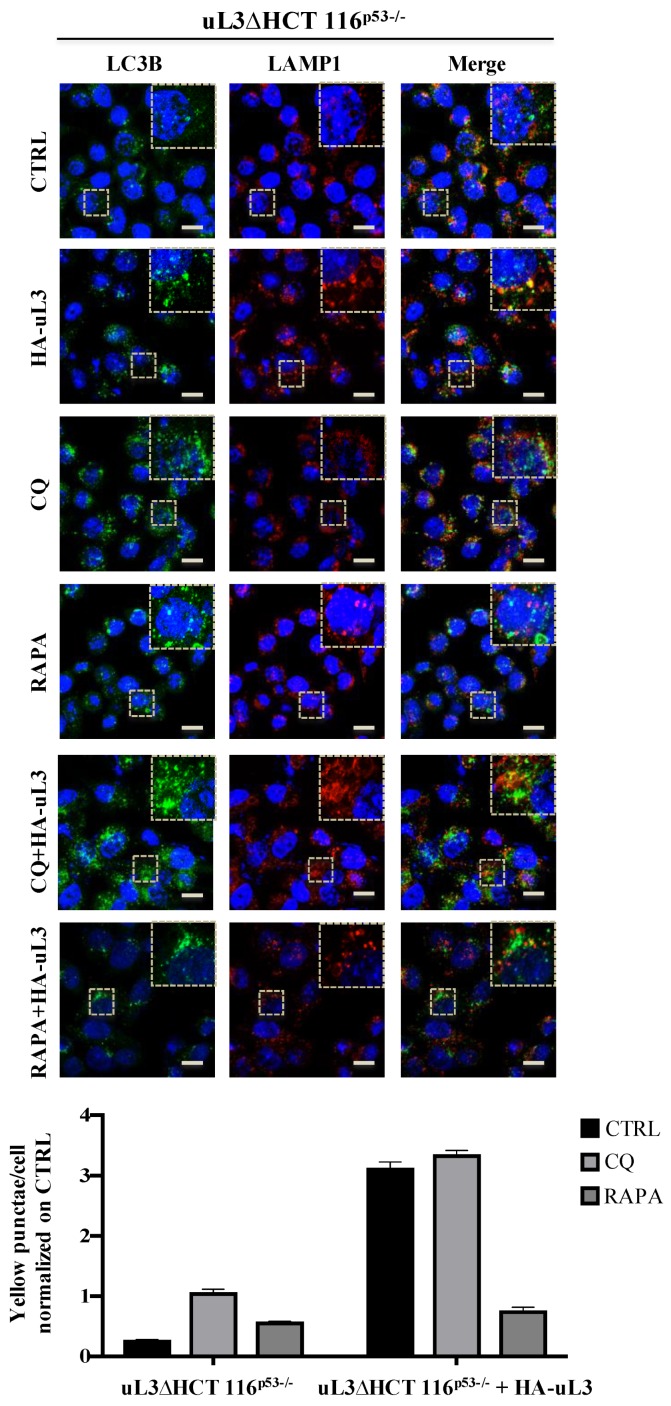
Depletion of uL3 enhances autophagic flux in HCT 116^p53−/−^ cells and the restoration of uL3 in uL3ΔHCT 116^p53−/−^ cells rescues this effect. Representative z-stack images of uL3ΔHCT 116^p53−/−^ treated with 25 µM CQ or 1 µM RAPA for 4 h or transiently transfected with 1 µg of pHA-uL3 for 24 h, alone or in combination. Cells were fixed and double stained with LC3B and LAMP1 antibodies. Nuclei were counterstained with Hoechst. Single-color fluorescence images of LC3B positive autophagosomes (green) and LAMP1 positive endosomes and/or lysosomes (red) are presented in the 1st and 2nd columns, respectively, and a merged view of these 2 proteins is shown in the 3rd column. Higher magnification views of the boxed area from the merged images are shown. Scal bar, 10 μm.

Figure 5 shows that in uL3 deleted cells, the absence of uL3 was associated to the increase of autophagic flux since LC3B puncta (green) and LAMP1 puncta (red), consistent with enhanced autophagosome and lysosome formation, respectively, were markedly increased compared to control cells, HCT 116^p53−/−^ cells. In uL3 deleted cells, CQ failed to inhibit autophagy (Figure 5). These results indicate a role of uL3 in CQ mediated inhibition of autophagy.

Of note, the restoration of uL3 in uL3∆HCT 116^p53−/−^ cells rescued the effect of uL3 on autophagy. In fact, the enforced expression of uL3 leads to an increase of yellow punctate structures (LC3B-LAMP1) to the proximity of the nucleus (Figure 5).

In uL3∆HCT 116^p53−/−^ cells transfected with pHA-uL3 and treated with CQ no significant difference were observed. Moreover, the rescue of uL3 in uL3∆HCT 116^p53−/−^ cells prevented the activation of autophagy by RAPA (Figure 5).

These data indicate that uL3 plays as inhibitor of autophagy acting on mTOR pathway.

Next, we assess the autophagic flux by Western blotting. LC3B has two isoforms, LC3B-I and LC3B-II; LC3B-I is cleaved and lipidated to form LC3B-II that is required for the formation of the autophagosome. Thus, the expression of LC3B-II is used as a marker to evaluate the autophagy since its level is proportional to the amount of autophagosomes in the cell.

We measured the abundance of LC3B-I and lipidated LC3B-II and the LC3B-II/I ratio by Western blotting in HCT 116^p53−/−^ and uL3∆HCT 116^p53−/−^ cells in condition of uL3 over-expression or CQ treatment.

**Figure 6:**
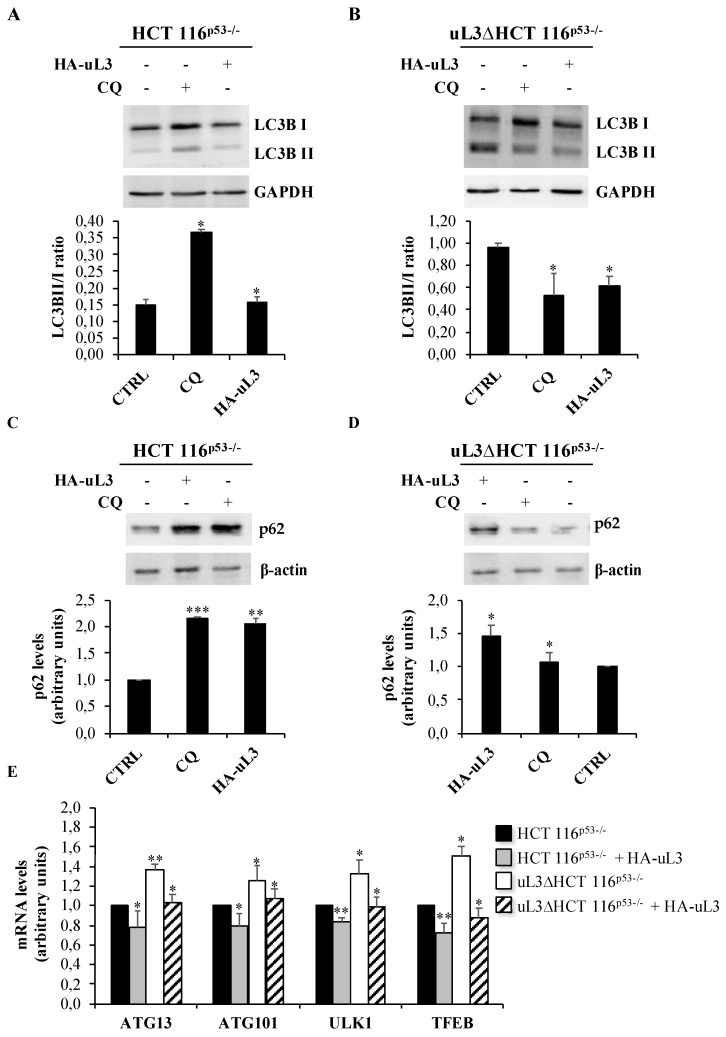
Effect of uL3 status on the conversion of LC3B and on the expression of autophagy-related genes. Representative immunoblotting showing LC3B protein conversion (**A**) and p62 protein levels (**C**) in HCT 116^p53−/−^cells and LC3B protein conversion (**B**) and p62 protein levels (**D**) in uL3ΔHCT 116^p53−/−^ cells treated or not with 25 µM CQ or transiently transfected with 1 µg of pHA-uL3. 24 h later, protein extracts from the samples were analyzed by Western blotting with antibodies against indicated proteins. GAPDH and β-actin were used as loading controls. Quantification of LC3B-II/I ratio is shown. Full-length blots are shown in Figures S7 and S8. (**E**) Total RNA from HCT 116^p53−/−^ and uL3ΔHCT 116^p53−/−^ cells, transfected or not with 1 µg of pHA-uL3, was subjected to RT-qPCR with primers specific for the indicated genes (Table 1). Quantification of signals is shown. Bars represent the mean of triplicate experiments; error bars represent the standard deviation. * *p* < 0.05, ** *p* < 0.01 vs. untreated cells set at 1.

As shown in Figure 6A, treatment of HCT 116^p53−/−^ cells with CQ caused a substantial increase in the amount of LC3B-II as well as the LC3B-II/I ratio. The increase in LC3B reflects a buildup of un-degraded autophagosomes and LC3B due to a lack of autophagosome fusion with lysosomes caused by CQ. Of note, in uL3∆HCT 116^p53−/−^ CQ failed to inhibit autophagy in accordance with the immunofluorescence experiments (Figure 6B).

The enforced expression of uL3 in HCT 116^p53−/−^ cells caused an increase of LC3B-I and a consequent decrease of LC3B-II/I compared to control, untransfected HCT 116^p53−/−^ cells, indicating the inhibitory role of uL3 on autophagy (Figure 6A).

These data are in line with the results of immunofluorescence experiments.

The restoration of uL3 in uL3∆HCT 116^p53−/−^ cells caused a decrease of LC3B-II/I indicating that autophagy induction that is associated in these cells to chemoresistance can be rescued by transfecting cells with a plasmid expressing uL3 (Figure 6B). These data were confirmed by the analysis of autophagy-mediated degradation of p62 (Figure 6C,D) indicating that autophagic flux is up-regulated in uL3∆HCT 116^p53−/−^ cells.

Next, we investigated whether the uL3 status can induce changes in transcript levels of some key autophagy related genes. To this aim, total RNA was extracted from HCT 116^p53−/−^ and uL3ΔHCT 116^p53−/−^ cells transfected or not with pHA-uL3 and analyzed by RT-qPCR with primers specific for ATG13, ATG101 and ULK1 (Table 1) that are essential components of the autophagy initiating ULK complex in higher eukaryotes [19]. We analyzed also the expression of Transcription factor EB (TFEB), a protein that promotes the expression of genes required for autophagosome formation, lysosome biogenesis and function [19]. Figure 6E shows that in HCT 116^p53−/−^ cells transfected with pHA-uL3 the expression level of all these genes resulted down-regulated while in absence of uL3 an opposite effect was observed. These data confirm the inhibitory role of uL3 on autophagy and the consequent up-regulation of this process in absence of uL3. Of note, the restoration of uL3 in uL3∆HCT 116^p53−/−^ cells rescued the effect of the absence of uL3 on autophagy. In fact, we observed a down-regulation of all the tested genes that is associated to a negative regulation of the autophagic process.

### 2.5. Effect of uL3 Restoration in uL3 Deleted Cells on Cell Cycle and Apoptosis

**Figure 7:**
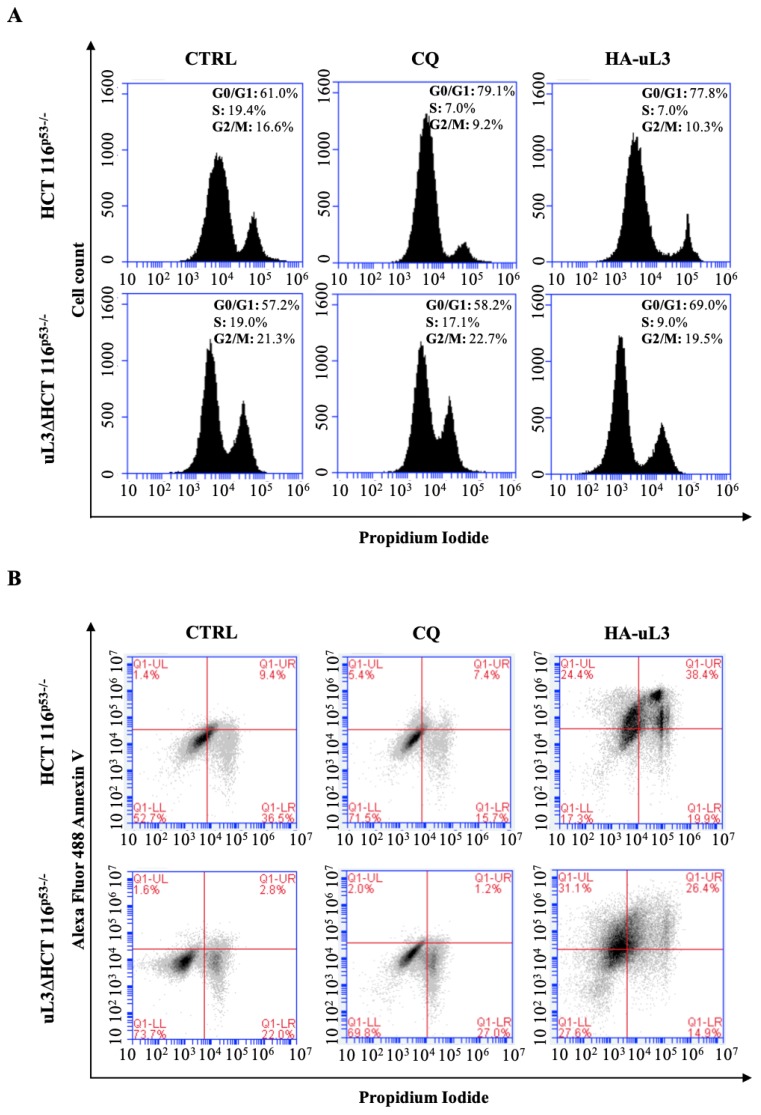
Effect of uL3 restoration in uL3ΔHCT 116^p53−/−^ cells on cell cycle and apoptosis. (**A**) HCT 116^p53−/−^ and uL3ΔHCT 116^p53−/−^ cells were transiently transfected with 1 µg pHA-uL3 or treated with 25 µM CQ. 24 h later, cells were stained with PI and analysed using FACS. Peaks representing histograms of cell numbers and percentages in G1, S, and G2/M phases are shown. (**B**) Representative flow cytometry dot blots with double Annexin V-Alexa Fluor 488/PI staining for HCT 116^p53−/−^ and uL3ΔHCT 116^p53−/−^ transiently transfected with pHA-uL3 or treated with CQ for 24 h.

With the aim of further examining the effect of uL3 restoration in uL3 deleted cells, alterations in the cell cycle distribution were analyzed. To this aim, HCT 116^p53−/−^ and uL3ΔHCT 116 ^p53−/−^ cells were transfected with pHA-uL3 or treated with CQ. 24 h later, cell cycle distribution was monitored by flow cytometry. As shown in Figure 7A, HCT 116^p53−/−^ cells treated with CQ resulted in an enlarged proportion of cells in G1 phase arrest (79%) while in uL3 deleted cells CQ failed to cause this effect confirming results from confocal microscopy and Western blotting experiments. As expected, upon uL3 transfection HCT 116^p53−/−^ cells were blocked in G1 phase (78%) [14]. Of note, the enforced expression of uL3 in uL3ΔHCT 116^p53−/−^ cells caused an increase of cells in G1 phase (70%) compared to the untransfected cells (Figure 7A).

Next, apoptosis was analyzed by Annexin V-Alexa Fluor 488/PI dual staining. As expected, we found that pHA-uL3 transfection in HCT 116^p53−/−^ cells significantly increased the percentage of late apoptotic cells (Annexin V^+^ and PI^+^) from 9% in control cells, untreated HCT 116^p53−/−^ cells, to 38% (Figure 7B). Furthermore, the enforced expression of uL3 in uL3ΔHCT 116^p53−/−^ cells caused an increase of apoptotic cells from 2.8% in absence of uL3 to 26.4% in cells transfected with pHA-uL3 (Figure 7B).

Figure S2 shows that uL3ΔHCT 116^p53−/−^ cells are resistant to Act D treatment.

All together these results strongly suggest that the ectopic expression of uL3 in cells devoid of uL3 and p53 induces a G1 cell cycle arrest associated to induction of apoptosis. Furthermore, these experiments confirmed the key role of uL3 in the regulation of CQ activity.

## 3. Discussion

In cancer therapy, the development of drug resistance is becoming a major challenge [27]. Studies on the mechanisms of cancer drug resistance have yielded important informations about how to overcome the resistance improving cancer chemotherapy. Drug resistance is a multi-factor involved process and recent studies have identified novel pathways involved in this process [27]. Increasing evidence suggest that tumor resistance to anticancer therapies can be correlated to induction of nucleolar stress and up-regulation of autophagy in different cancer cells [20,28]. In line with this evidence, data presented in this paper demonstrate that alteration of uL3 expression levels in HCT 116^p53−/−^ cells induced nucleolar stress (Figure 1 and Figure 2). Furthermore, uL3 overexpression inhibited autophagy (Figure 4 and Figure 6A,C) while the absence of this protein in uL3ΔHCT 116^p53−/−^ cells [10] was associated to the upregulation of autophagy (Figure 5 and Figure 6B,D).

Nucleolus, the site of ribosome biogenesis, is recently considered as central hub in the cellular stress response [29,30]. Nucleolus can favor neoplasia since many proteins which are related to ribosome production as r-proteins perform non-ribosomal functions and can be engaged in malignancy, including transformation, cancer development and drug resistance [31]. On the other hand, the nucleolus can have anticancer activity when ribosome biosynthesis is impaired by stressors including intentional therapeutic action, which is followed by a protective response [31].

Perturbation in pre-rRNA processing or alteration in the r-protein content impair ribosome biogenesis and trigger the nucleolar stress response [4]. In this condition a sub set of r-proteins translocate to the nucleoplasm where activate p53 leading to cell cycle arrest and/or apoptosis [4]. Beside its function on cell cycle regulation and apoptosis, p53 can also inhibit mTOR pathway via activation of AMPK or by increasing PTEN expression whit consequent activation of autophagy [32]. Nucleolar stress can also be activated in cancer cells expressing mutant p53 since novel pathways p53-independent have been discovered that activate cell cycle arrest and/or apoptosis [33]. uL3 is not only a ribosome-building element but also a key element in the activation of nucleolar stress pathway p53-independent in response to chemotherapeutics interfering with nucleolar function as Act D [4]. In the presence of Act D, we found up-regulation of uL3 expression and its translocation from the nucleolus in the nucleoplasm where it can exert its extra-ribosomal functions [12].

Here, we go more in depth in understanding the mechanism by which uL3 is able to activate nucleolar stress pathway. Our results demonstrated that uL3 inhibited RNA pol I function with consequent down-regulation of 47S pre-rRNA production (Figure 1B and Figure 2). Furthermore, analysis of rRNA processing pathway indicated that cells over-expressing uL3 showed also defects in the processing of 47S pre-rRNA with consequent accumulation of 45S, 30S and mature rRNA forms 28S an 18S. Deletion of uL3 caused a significant enrichment of 36S and 45S pre-rRNA while the levels of 28S, 18S, and 5,8S resulted only slightly affected (Figure 1B). These data suggest that altered expression levels of uL3 cause nucleolar stress but the effect in term of cell response may be different depending on the intracellular amount of uL3. In fact, high levels of uL3 impair rRNA processing leading to cell death and inhibition of autophagy (Figure 1, Figure 2, Figure 4, Figure 6A,C,E, and Figure 7B) whereas silencing of uL3 associated to defects of rRNA maturation leading to activation of autophagic flux (Figure 1, Figure 2, Figure 5, and Figure 6B,D,E).

Deregulation of r-protein expression occurs in many types of cancers. In particular, mutations and deletions of r-proteins increase susceptibility to various diseases including cancers [34].

We have previously demonstrated that silencing of uL3 in colon and lung cancer cells is associated to chemoresistance and that the ectopic expression of uL3 re-sensitive these cells to chemotherapeutic treatment [9,17]. In order to elucidate the role of uL3 in the activation of nucleolar stress pathway associated to chemoresistance we have used RNA seq analysis to identify cellular processes, genes and pathways that are differentially expressed in HCT 116^p53−/−^ cells in presence or absence of uL3 and in condition of nucleolar stress activated by Act D. Numerous processes underwent dramatic changes in dependence of the uL3 status, many associated with major pathways and processes controlling metabolism and autophagy such as PI3K_AMPK signaling, IL6_JAK-STAT3 complex, mTORC1, WNT-Beta-Catenin, or processes controlling cell fate as EMT, DNA damage, apoptosis, cell cycle (Figures S3 and S4). Of interest, RNA seq analysis of HCT 116^p53−/−^ cells treated with Act D compared to untreated cells revealed up-regulation of mTORC1, IL6_JAK_STAT3 while in uL3ΔHCT 116^p53−/−^ we found a down-regulation of mTORC1, PI3K_AKT_mTORC1, WNT_Beta_Catenin suggesting a role of uL3 as inhibitor of autophagy.

In recent years, some findings suggested that an intricate relationship between autophagy and EMT exists in cancer [35]. According to its dual role in tumorigenesis, the effect of autophagy on EMT appears controversial and strictly dependent on the cellular type and on the stimulus employed for activating or inhibiting autophagy.

In our experimental model, we found that the lower expression of uL3 associated to autophagy activation and EMT phenotype.

Autophagy is a crucial cytoprotective strategy essential for the cellular homeostasis affording, among other functions, degradation of intracellular components such us protein aggregates, organelle, soluble protein and other cellular elements [19].

Various cellular stressors can activate either autophagic response than nucleolar stress including nutrient deprivation, low energy, hypoxia, UV irradiation, chemical compounds and diverse classes of anticancer drugs [18]. In mammalian, autophagy is regulated by a complex network of signaling pathways that finally converges in PI3K_mTORC1 pathway representing the principal regulator of autophagy machinery that is constituted by more than 30 autophagy-related (ATG) proteins [19]. Considering the essential role of autophagy in cellular homeostasis it is expected that alteration in this process is associated with the development of different disease including cancer [18].

The role of autophagy in cancer is complex and strictly dependent on cell context [20]. There are different examples of cancer types in which autophagy has a tumor suppressive role as in PTEN and Beclin 1 knockouts and others in which autophagy sustains tumor growth in these cases the inhibition of autophagy represents a promising therapeutic approach [36].

In view of the results from transcriptomic analysis, we hypothesized that the chemoresistance observed in uL3 deleted colon cancer cells [9,17] might depend on the higher autophagic activity displayed by this cell population. Analysis of the autophagic flux by confocal microscopy demonstrated that overexpressing uL3 in HCT 116^p53−/−^ cells markedly suppressed autophagic flux, as the number of LC3B dots and the accumulation of LC3B-II protein were decreased. In these cells, RAPA failed to exert its positive effect on autophagy while inhibition of autophagy was observed when these cells were treated with CQ. Of interest, in uL3ΔHCT 116^p53−/−^ cells the autophagic flux was most markedly elevated and CQ failed to inhibit autophagy. The rescue of uL3 in uL3∆HCT 116^p53−/−^ cells prevented the activation of autophagy by RAPA. All together, these results indicate that uL3 acts as inhibitor of autophagy. The role of uL3 in autophagy was also demonstrated by the analysis of transcript levels of some key autophagy related genes. We found that the ectopic expression of uL3 in HCT 116^p53−/−^ cells induced reduction of mRNAs coding for protein components of autophagy initiating ULK complex, while in uL3ΔHCT 116^p53−/−^ cells increased levels of these proteins were detected. Of interest, the enforced expression of uL3 in uL3ΔHCT 116^p53−/−^ cells rescued ULK complex mRNA up-regulation (Figure 6E). These data confirm the ability of uL3 to act as a repressor of autophagy. Beside autophagy, uL3 is known to regulate cell cycle and to induce apoptosis in colon cancer cells [12,14]. In this paper we report that in HCT 116^p53−/−^ cells the percentage of cells in G0/G1 increase in condition of uL3 over expression as well as after treatment with CQ. Of note, the treatment of uL3ΔHCT 116^p53−/−^ cells with CQ had no effect on cell cycle and this is in line with results from confocal microscopy confirming the key role of uL3 in CQ activity. These data give more insight into the mechanism of action of CQ, a well known autophagic inhibitor, which has been demonstrated to enhance 5-FU anticancer activity in colon cancer cells both in vitro and in vivo [37,38]. The overexpression of uL3 in uL3ΔHCT 116^p53−/−^ completely rescued cell cycle arrest and apoptosis mediated by uL3.

In conclusion, these observations imply a possibility that depletion of uL3 may increase the resistance of colon cancer cells to drug treatment through autophagy induction, whereas the restoration of uL3, through the inhibition of autophagy, may drive uL3ΔHCT 116^p53−/−^ cells to cell death by apoptosis.

Our findings strongly suggest that nucleolar stress and autophagy are tightly coupled in our model of colon cancer in order to enhance the anticancer effect of chemotherapeutic drugs.

Thus, our study supports the notion that autophagy inhibitors are beneficial to chemotherapy in colon cancer cells.

## 4. Material and Methods

### 4.1. Cell Cultures, DNA, Transfections and Drug Treatments

HCT 116^p53−/−^ cells and uL3ΔHCT 116^p53−/−^, a cell line derived from HCT 116^p53−/−^ cell and stably silenced for uL3 [10], were cultured at 37 °C in a 5% CO_2_ humidified atmosphere and grown in Dulbecco’s Modified Eagle’s Medium (DMEM) supplemented with 10% fetal bovine serum (FBS), 2 mM L-glutamine and penicillin-streptomycin 50U/mL.

Plasmid transfections (1 μg of pHA-uL3) were performed in cells as previously described [39]. Drug treatments were performed by adding to cells Act D, CQ, and RAPA (Sigma-Aldrich, St. Louis, MO, USA).

### 4.2. RNA Extraction and RT-qPCR

Total RNA was extracted from HCT 116^p53−/−^ and uL3ΔHCT 116^p53−/−^ cells treated or not with Act D and transfected or not with pHA-uL3 using the miRNeasy kit (Qiagen, Hilden, Germany) according to the manufacturer’s instructions and quantified using the ND-8000 spectrophotometer (NanoDrop Technologies, Wilmington, DE, USA). RNA integrity was assessed using an RNA 6000 Nano chip on a Bioanalyzer (Agilent Technologies, La Jolla, CA, USA).

Total RNA was retrotranscribed as previously reported [40] and qPCR was carried out using SensiFAST SYBER^®^ No-ROX kit (Bioline, London, UK). The primers are indicated in Table 1. The comparative Ct method was used to calculate the relative abundance of the mRNA and compared with that of β-actin expression [41].

### 4.3. Library Preparation and Deep Sequencing

For RNA-seq analysis, libraries were prepared according manufacturer’s instructions (TruSeq RNA Sample Preparation kit, Illumina, San Diego, CA, USA) starting from 4 μg of total RNA. Quality control of library templates was performed using a High Sensitivity DNA Assay kit (Agilent Technologies, La Jolla, CA, USA) on a Bioanalyzer (Agilent Technologies, La Jolla, CA, USA). The Qubit quantification platform (Qubit 2.0 Fluorometer, Life Technologies, Carlsbad, CA, USA) was used to normalise samples for the library preparation. Using multiplexing, up to 6 samples were combined into a single lane to yield sufficient coverage. The sequencing was carried out in collaboration with the Next Generation Sequencing (NGS) Facility at TIGEM. Cluster generation was performed on Flow Cell v3 (TruSeq PE Cluster Kit v3; Illumina San Diego, CA, USA) using cBOT. Libraries were sequenced by a paired-end chemistry on an NovaSeq6000 platform. Each library was loaded at a concentration of 8 pM, which was previously established as optimal. An average yield of ~4.5 Mb was obtained per sample. The data have been deposited in NCBIs Gene Expression Omnibus (GEO) [42]. GEO accession number is GSE145807.

### 4.4. Computational Analysis of Deep Sequencing Data

A data analysis was performed using the pipeline already established at the Bioinformatics and Statistics Core Facility at TIGEM [43]. Briefly, the reads were trimmed to remove adapter sequences and low-quality ends and reads mapping to contaminating sequences (e.g., ribosomal RNA, phIX control) were filtered-out. Reads were aligned and assigned to Human ENSEMBLE transcripts and genes (hg38 reference) by using RSEM version 1.2.25 with standard parameters [44]. The threshold for statistical significance chosen was False Discovery Rate (FDR) < 0.05. The Gene set enrichment analysis (GSEA) [45] was then performed restricting the output to the collection of “hallmark” gene sets [46] part of the Molecular Signatures Database (MSigDB v7.0). The threshold for statistical significance chosen in the GSEA was False Discovery Rate (FDR) < 0.25.

### 4.5. Nuclear Run-On

Nuclear run-on was performed as previously reported [47]. Nuclei from approximately from 10^8^ cells were incubated in a buffer containing 10 mM Tris, pH 7.5; 10 mM MgCl2; 300 mM KCl; 0.5 mM each of ATP, CTP, and GTP; 15 μL [α^−32^P]UTP [3000 mCi/mL]), and incubated at 30 °C for 30 min. Labeled RNA was extracted by using Trizol reagent (Invitrogen, Life Technologies, Carlsbad, CA, USA) according to the manufacturer’s specifications. Plasmid DNAs were immobilized on a GeneScreen Plus membrane using a dot-blot apparatus. pCDNA3 plasmid was used as control. pCDNA3 was spotted on membrane and incubated with ^32^P-labeled RNA from untreated HCT 116^p53−/−^, HCT 116^p53−/−^ cells transfected with 1µg of pHA-uL3 and HCT 116^p53−/−^ cells treated with Act D. Observed signals for pCDNA3 in all samples represent the background signal. Intensity of signals was normalized on pcDNA3 signal. Hybridization was carried out as indicated in GeneScreen Plus manual.

### 4.6. Immunofluorescence

HCT 116^p53−/−^ and uL3ΔHCT 116^p53−/−^ cells seeded on slides were fixed for 10 min with Methanol (Sigma-Aldrich, St. Louis, MO, USA), followed by incubation with blocking–permeabilization solution: 0.5% BSA, 0.1% saponin, and NH_4_Cl 50 mmol/L in phosphate buffered saline for 30 min. Primary antibodies anti-LC3B (Novus Biologicals, Centennial, CO, USA) and anti-LAMP1 (Developmental Studies Hybridoma Bank) were diluted in blocking–permeabilization solution and added to the cells for 1 h. Then, secondary antibodies were incubated for 45 min. Lastly, cells were counterstained with Hoechst (Thermo Fisher Scientific, Uppsala, Sweden). Samples were examined under a confocal microscope (Zeiss LSM 700; Carl Zeiss AG, Jena, Germany) equipped with 40x and 63× 1.4 NA oil objective. Colocalization between specific markers was quantified using Zeiss Zen 2012 software.

### 4.7. Western Blot Analysis

A Western blotting analysis was performed as previously reported [48]. The membranes were challenged with anti-POLR1B (Abcam, Cambridge, UK), anti-LC3B, anti-p62, anti-HA, anti-GAPDH and anti-β-actin (Cell Signaling Technology, Danvers, MA, USA). Proteins were visualized with enhanced chemiluminescence detection reagent according to the manufacturer’s instructions (Elabscience^®^, Houston, TX, USA).

### 4.8. Cell Cycle Analysis

HCT 116^p53−/−^ cells and uL3ΔHCT 116^p53−/−^ cells were seeded into 35 mm tissue culture plates at a confluency about 50-60% (5 × 10^5^ cells). Then, cells were starved overnight treated with CQ (25 μM) or transiently transfected with pHA-uL3 (1 μg) for 24 h. After treatment, the cells were harvested and centrifuged at 400 g for 5 min, washed once with cold PBS and resuspended and fixed by adding 0.5 mL ice-cold 70% ethanol dropwise. Then, the cells were incubated on ice overnight. The cells were spun down and washed twice with PBS (Dulbecco’s Phosphate-Buffered Saline, Sigma-Aldrich, St. Louis, MO, USA). They were resuspended in a 500 μL of PBS and incubated with 50 μg/mL PI (Propidium Iodide, Sigma-Aldrich, St. Louis, MO, USA) for 30 min protected from light. Cell cycle distribution was analyzed using BD Accuri C6 Plus flow cytometer (BD Biosciences, San Jose, CA, USA).

### 4.9. Cell Death Assay

HCT 116^p53−/−^ and uL3ΔHCT 116^p53−/−^ cells (5 × 10^5^) were seeded in 6-well plate per well, starved overnight and treated with CQ (25 μM) or transiently transfected with pHA-uL3 (1 μg) for 24 h. The cells were washed with PBS, harvested by trypsinization and washed twice with PBS. The cells were then stained with Propidium Iodide (PI) and Annexin V Alexa Fluor^®^ 488 using Tali^®^ Apoptosis Kit (Life Technologies, Carlsbad, CA, USA) according to manufacturer’s instruction. Briefly, cells were re-suspended with 1× binding buffer at a density of 1 × 10^6^ cells/mL. Then, PI and Annexin V Alexa Fluor^®^ 488 (5 μL) was added to cell suspension (100 μL) before further incubation for 20 min at RT in the dark. Stained cells were diluted with 1× binding buffer and analyzed by BD Accuri C6 Plus flow cytometer (BD Biosciences, San Jose, CA, USA). The percentage of Annexin V+/PI− (early apoptosis), Annexin V^+^/PI^+^ (late apoptosis), and Annexin V^−^/PI^+^ (necrosis) cells was analyzed on the basis of manufacture’s instruction. The data are represented as rate of total apoptotic cells with both early and late apoptotic rate indicated.

### 4.10. Statistical Analysis

Statistical analysis was performed as previously reported [49].

## Figures and Tables

**Table 1:** Sequence of oligonucleotides used in RT-qPCR analysis.

Gene	Sequence
47S	Forward: 5′–GCTGACACGCTGTCCTCTG–3′ Reverse: 5′–ACGCGCGAGAGAACAGCAG–3′
45S	Forward: 5′–GCCTTCTCTAGCGATCTGAGAG–3′ Reverse: 5′–CCATAACGGAGGCAGAGACA–3′
36S	Forward: 5′–GCGGAGGTTTAAAGACCC–3′ Reverse: 5′–CCAGACGAGACAGCAAAC–3′
32S	Forward: 5′–GTCAGCGGAGGAGAAGAA–3′ Reverse: 5′–CTCGATCAGAAGGACTTGG–3′
30S	Forward: 5′–CCTCTGACGCGGCAGACAGC–3′ Reverse: 5′–CTCCAGGAGCACCGCAAGGG–3′
18S precursors	Forward: 5′–GTTCAAAGCAGGCCCGAGCC–3′ Reverse: 5′–AGCGGCGCAATACGAATGCC–3′
28S	Forward: 5′–CAGGGGAATCCGACTGTTTA–3′ Reverse: 5′–ATGACGAGGCATTTGGCTAC–3′
18S	Forward: 5′–AAACGGCTACCACATCCAAG–3′ Reverse: 5′–CCTCCAATGGATCCTCGTTA–3′
5.8S	Forward: 5′–CTCTTAGCGGTGGATCACTC–3′ Reverse: 5′–GACGCTCAGACAGGCGTAG–3′
ATG13	Forward: 5′–GACCTTCTATCGGGAGTTTCAG–3′ Reverse: 5′–GGGTTTCCACAAAGGCATCAAAC–3′
ATG101	Forward: 5′–CCCAGGATGTTGACTGTGAC–3′ Reverse: 5′–ACATCTGCCCCAGCCCATCG–3′
β-actin	Forward: 5′–CCAACCGCGAGAAGATGA–3′ Reverse: 5′–CCAGAGGCGTACAGGGATAG–3′
TFEB	Forward: 5′–CAAGGCCAATGACCTGGAC–3′ Reverse: 5′–AGCTCCCTGGACTTTTGCAG–3′
ULK1	Forward: 5′–CTGGTCCTCTTGCTTCCGTC–3′ Reverse: 5′–ACACCAGCCCAACAATTC–3′

## Supplementary Materials

The following are available online at https://www.mdpi.com/1422-0067/21/6/2143/s1.
