# Supplementary material for: Role of uL3 in the Crosstalk between Nucleolar Stress and Autophagy in Colon Cancer Cells
Source: Int J Mol Sci. 2020 Mar 20;21(6):2143. doi: 10.3390/ijms21062143 (PMC7139652; doi:10.3390/ijms21062143)
Supplement: Supplementary file 1 [file ijms-21-02143-s001.zip › Supplementary Materials/Figures S1-S9.pdf]

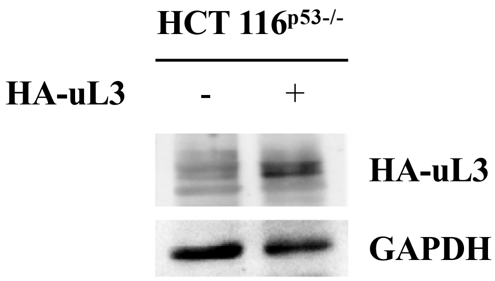

Figure S1. Trasfection efficiency of pHA-uL3 evaluated by western blotting. HCT 116$^{p53-/-}$ cells were transiently transfected with 1 μg of pHA-uL3. 24 h later, cells were lysated and protein extracts from the samples were analyzed by western blotting with antibodies against uL3 and GAPDH as loading control. Full-length blots are presented in Figure S9.

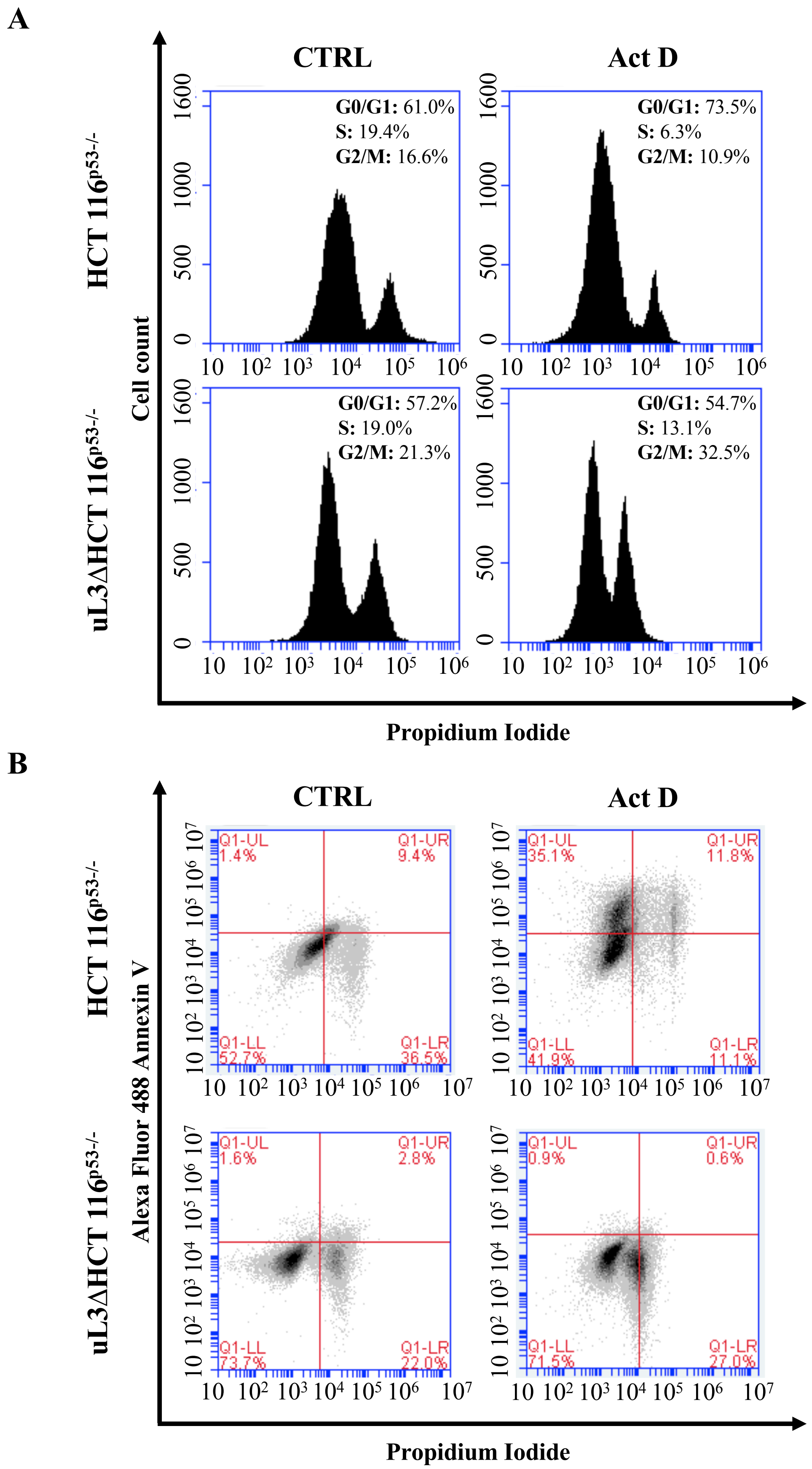

Figure S2. Effect of Act D treatment on cell cycle and apoptosis in uL3ΔHCT $116^{p53-/-}$ cells. (**A**) HCT $116^{p53-/-}$ and uL3ΔHCT $116^{p53-/-}$ cells were treated with 5 nM Act D. 24 h later, cells were stained with PI and analysed using FACS. Peaks representing histograms of cell numbers and percentages in G1, S, and G2/M phases are shown. (**B**) Representative flow cytometry dot blots with double Annexin V-Alexa Fluor 488/PI staining for HCT $116^{p53-/-}$ and uL3ΔHCT $116^{p53-/-}$ treated with 5 nM Act D for 24 h.

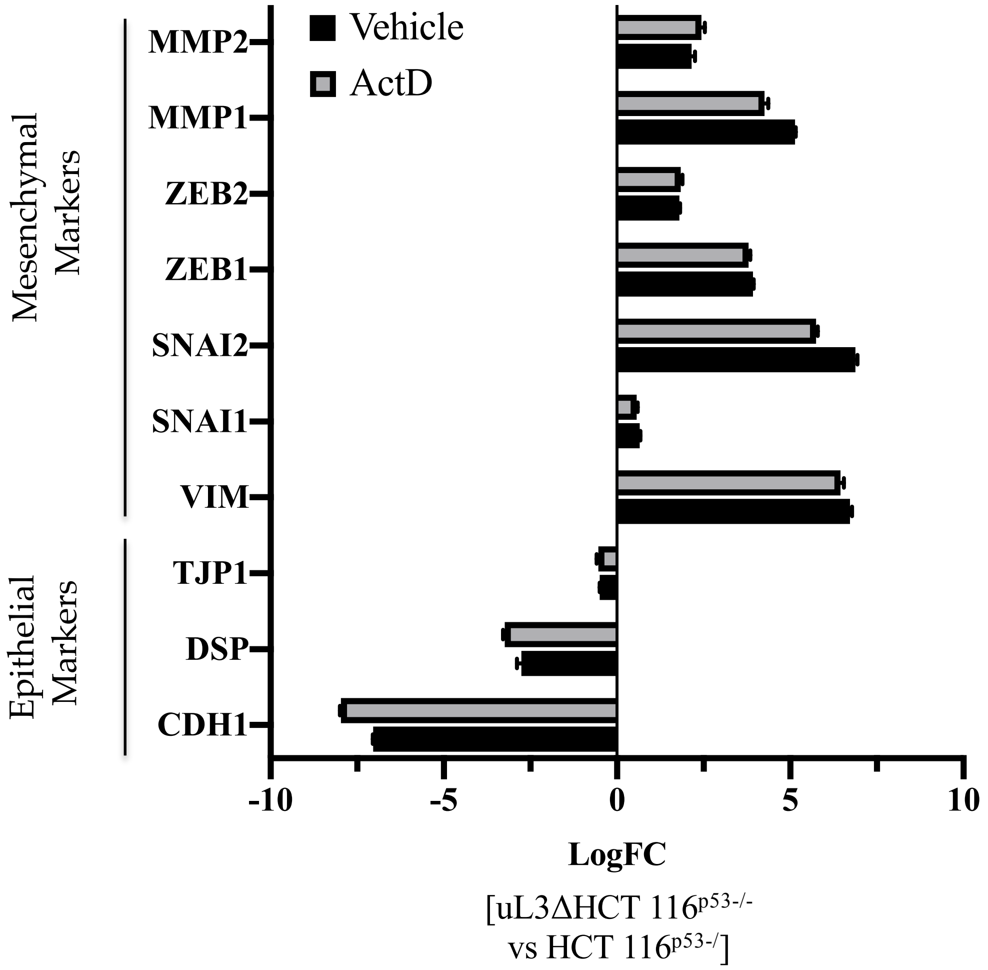

Figure S3. Epithelial-mesenchymal transition (EMT) related differential expressed genes between uL3ΔHCT $116^{p53-/-}$ and HCT $116^{p53-/-}$ cells treated or not with 5nM Act D for 24 h by RNA-seq analysis. Mesenchymal markers: MMP2, MMP1, ZEB2, ZEB1, SNAI2, SNAI1, VIM; epithelial markers: TJP1, DSP, CDH1; x-axis represents log 2 fold change of expressed genes vs average expression normalized to log 2 scale for each genes.

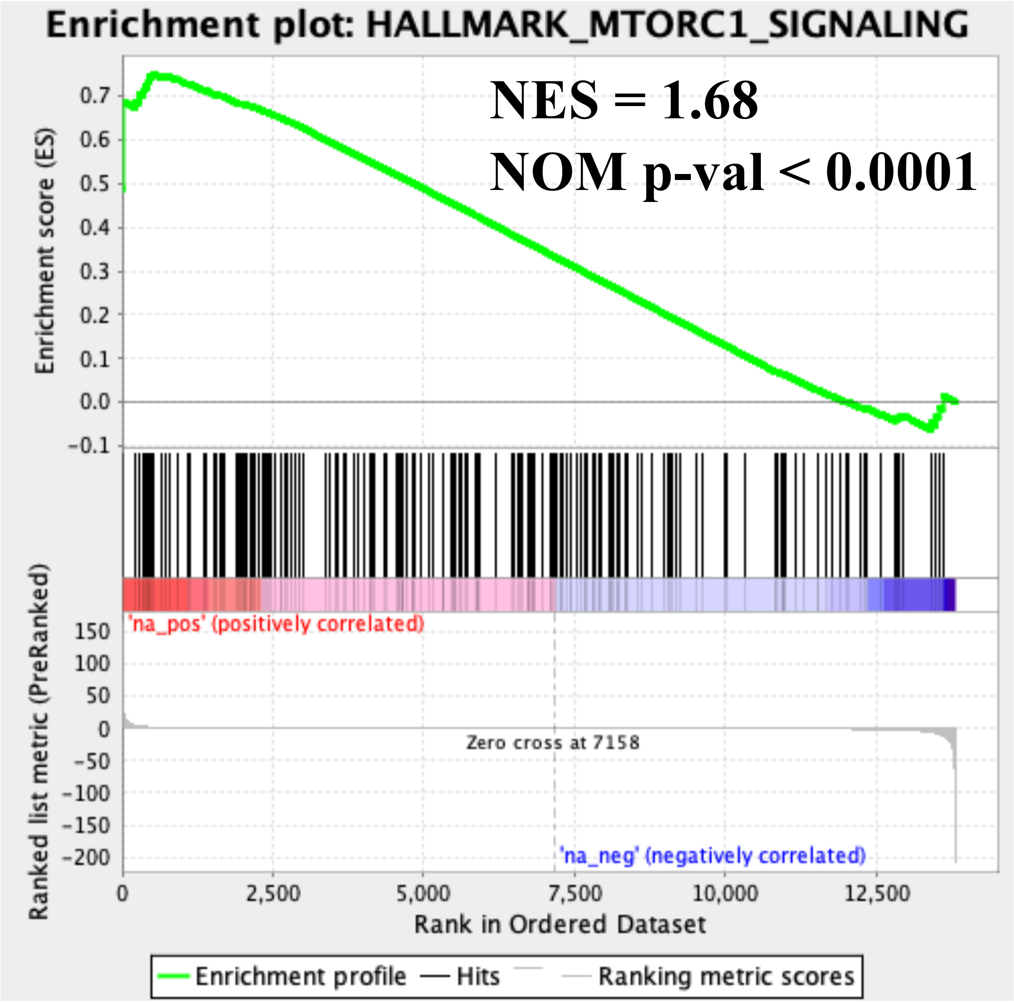

Figure S4. mTORC pathway regulated by Act D treatment in HCT 116$^{p53-/-}$ cells. The GSEA (Gene Set Enrichment Analysis) plots showing MTORC1_SIGNALING hallmark gene set in HCT 116$^{p53-/-}$ cells treated with Act D. The normalized enrichment scores (NES) and p value were indicated.

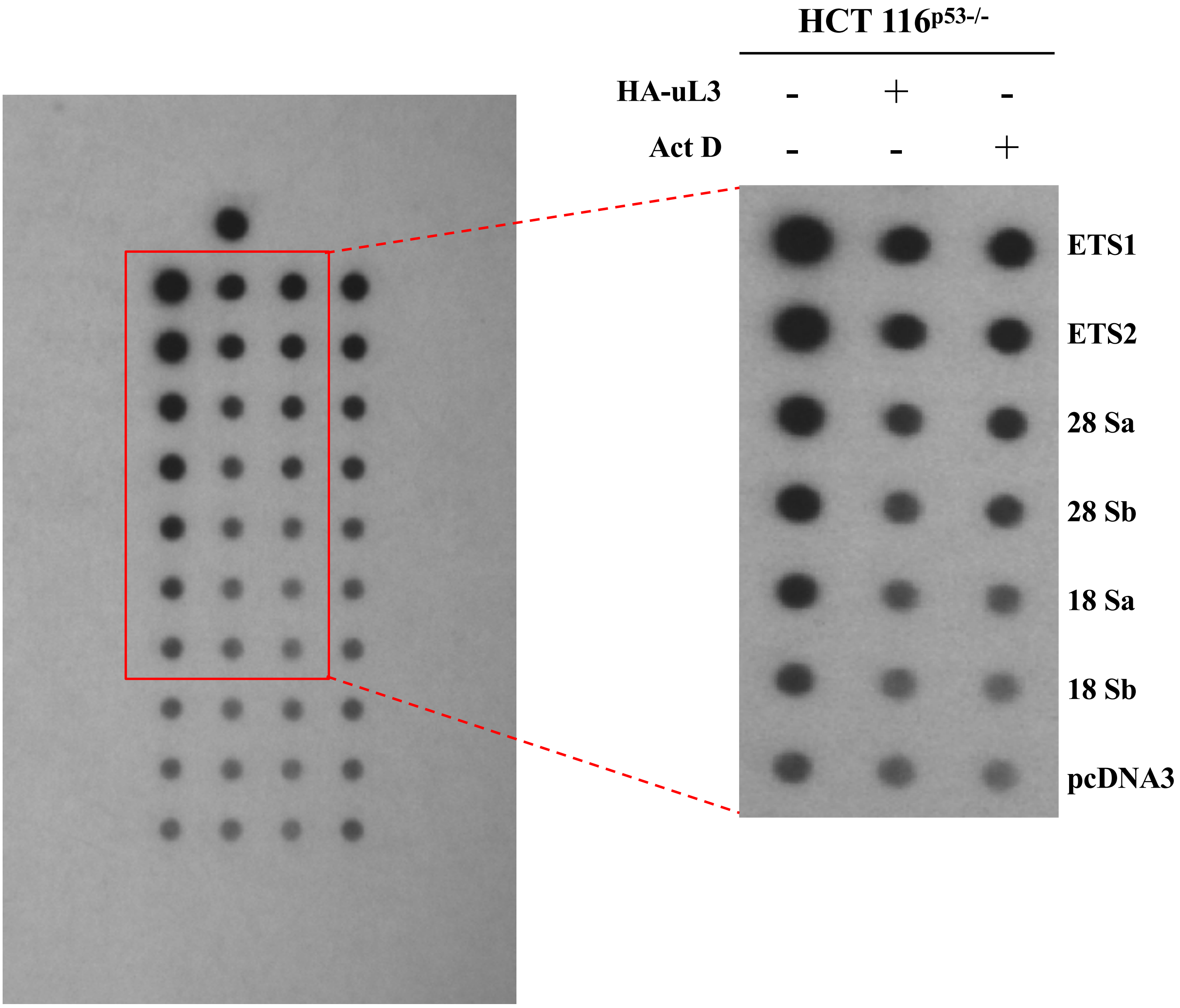

Figure S5. Full length blot of Figure 2B.

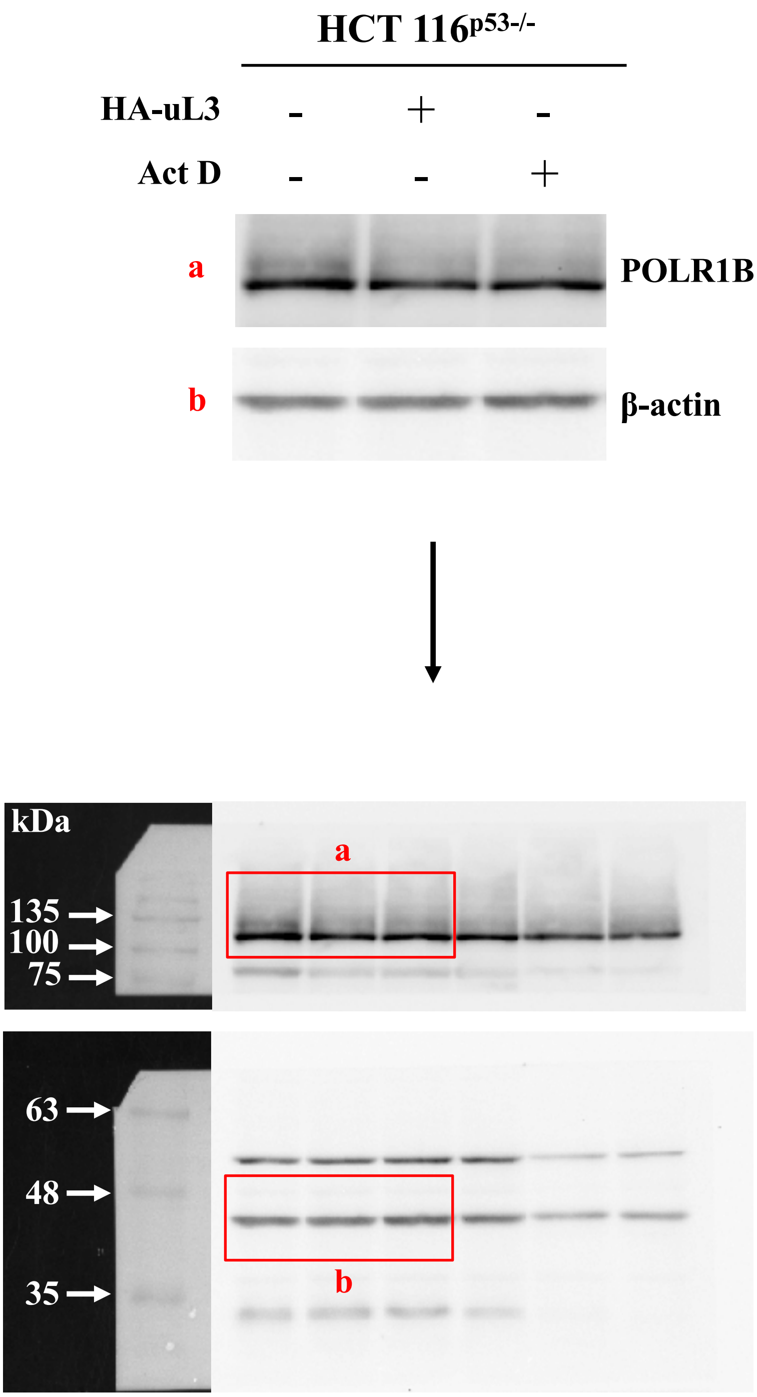

Figure S6. Full length blots of Figure 2C.

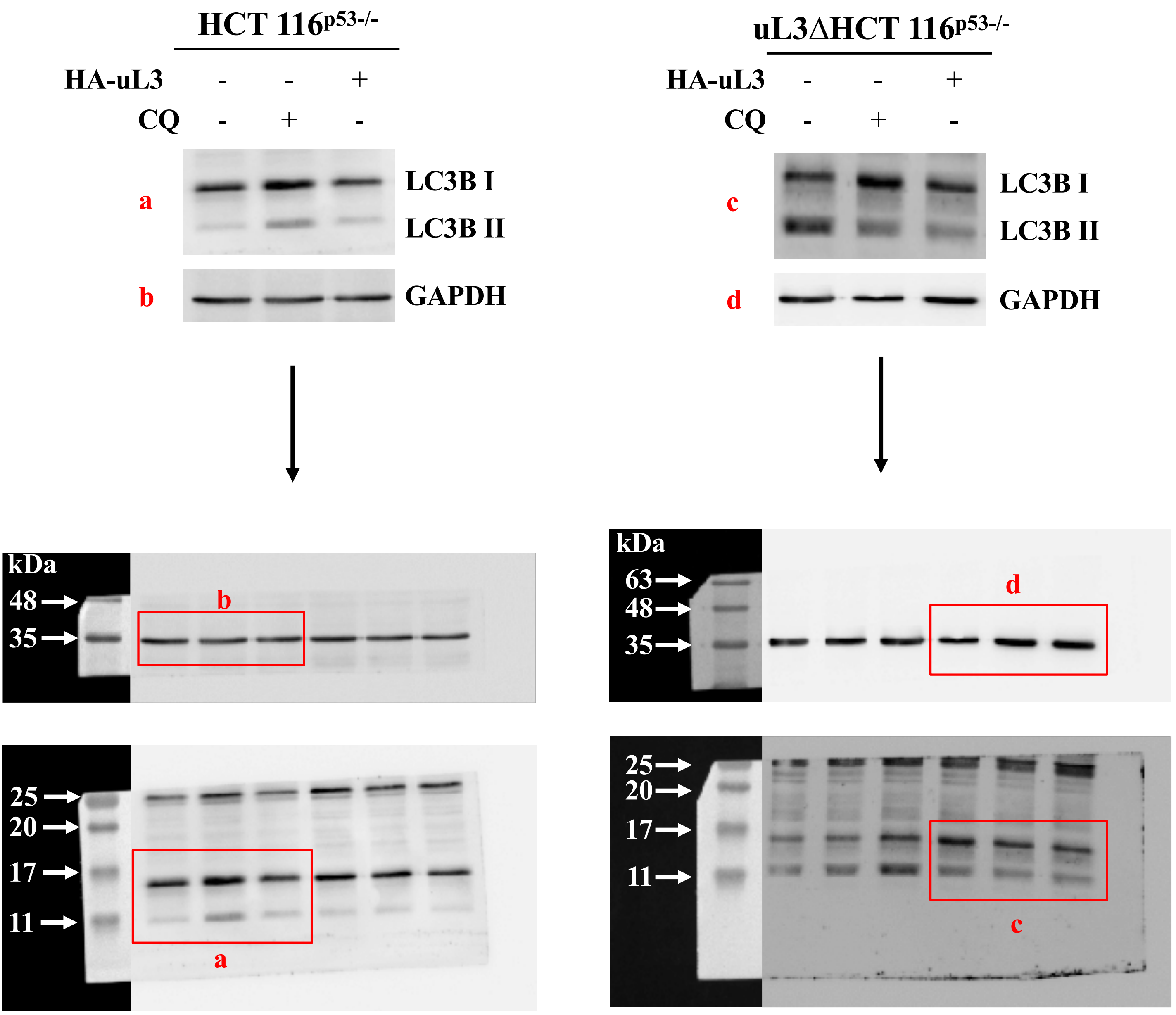

Figure S7. Full length blots of Figures 6A and 6B.

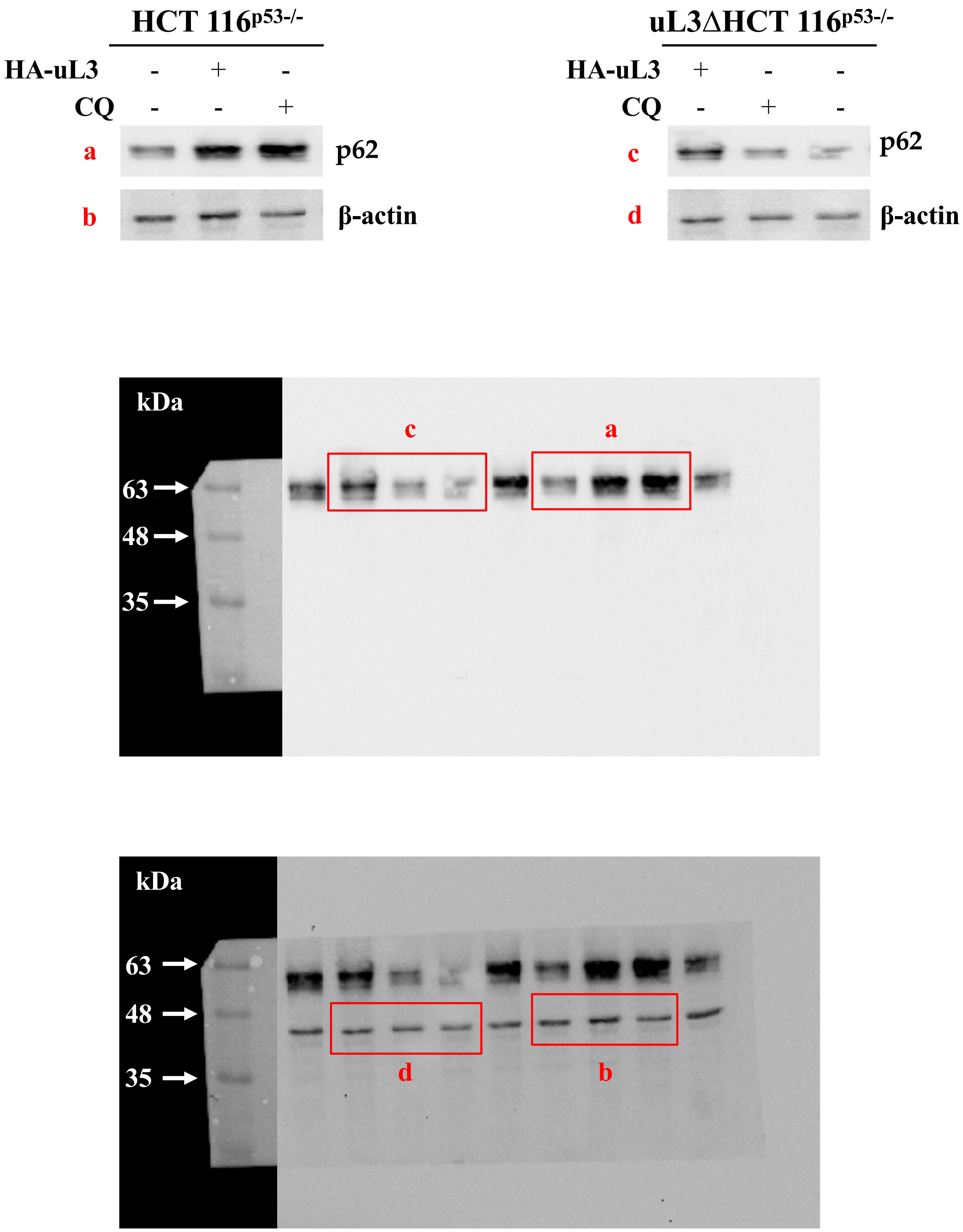

Figure S8. Full length blots of Figures 6C and 6D .

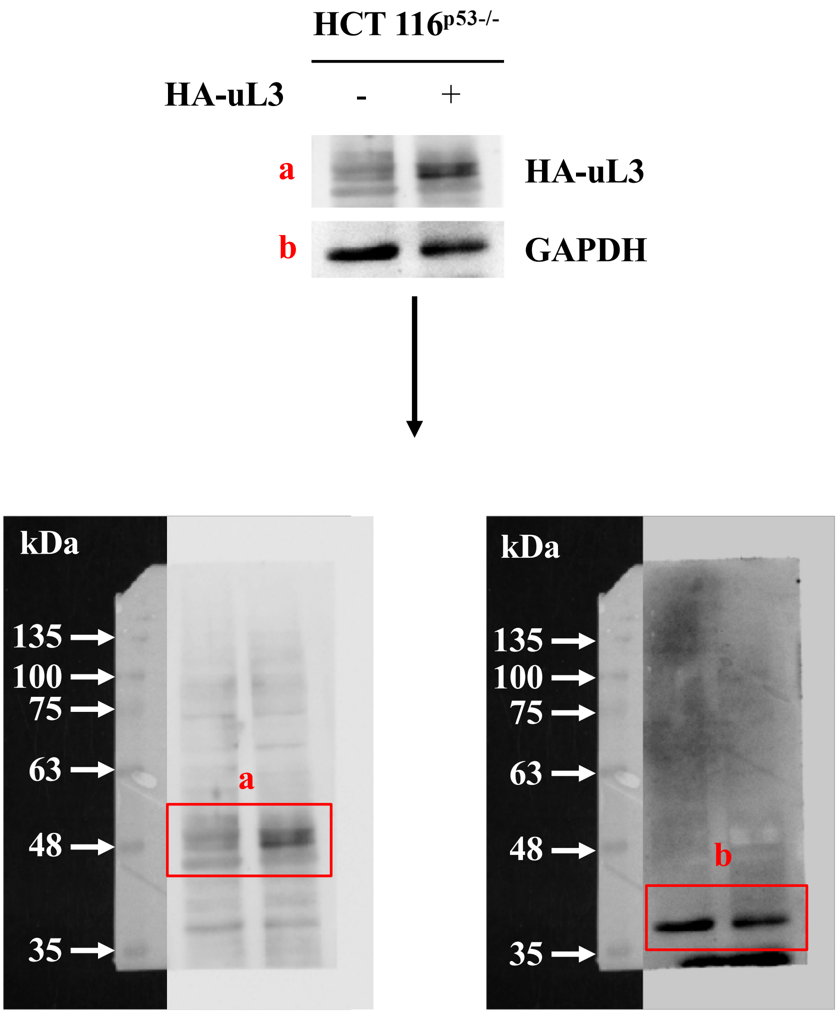

Figure S9. Full length blots of Figure S1.
